# Poisons and Poisoners

**Published:** 1892-04-30

**Authors:** 


### Poisons and Poisoners.

STRYCHNIA—DR. PALMER.

It is to be regretted that in dealing with poisons and poisoners we have so often to hold up members of the medical profession as criminals. But, if the doctors will only take it so, this is a left-handed compliment. They show skill and ingenuity, they arouse interest which the more illiterate criminal who wishes to get rid of wife or creditor, and buys a shilling packet of vermin killer to that end, never does; they are scientifically and pathologically worth dealing with. Therefore of the various cases recorded of poisoning by strychnia we select that of William Palmer, accused of poisoning a betting companion, John Parsons Cook.

Had he not had betting companions Palmer would never have been a poisoner. But when he set up in practice at Rugeley, in Staffordshire, he not only launched into an extravagant style of living, but bought and ran race-horses. As these extravagances embarrassed him he resorted to various shifts to raise money. He insured his wife's life in his own favour for the sum of £13,000, and the wife died within nine months. He then tried, but failed, to insure the life of his brother Walter, a confirmed drunkard, for no less than £80,000. Failing in this—for the insurance offices got a hint that his wife had died suspiciously soon after her life was insured—he tried to effect an insurance on the life of one, Bate, a decayed farmer, whom he represented to be a prosperous country gentleman. The insurance company sent a detective to see the country gentleman, who was found hoeing turnips, and declined to grant the insurance. Thus baffled, Palmer, to use his brother's euphemistic words, "infringed the commercial code of his country"—in short, committed forgery. He wrote his mother's name on bills amounting to £2,000, and when, during his imprisonment, he was brought up as witness in an action taken upon these, he put the guilt of the forgery on his dead wife. He was, in short, in the direst straits for money and was evidently unscrupulous as to how it was obtained.

Cook, the victim, was a rather foolish young man who, on inheriting a small fortune had thrown up his work as a solicitor and gone on the turf. It was through this that he became acquainted with Palmer, with whom he joined in some racing transactions. He lent his name to Palmer on a bill for £200, and was left to pay it. Palmer succeeded also in discounting a bill of Cook's for £500, had the money sent to Doncaster Post Office, obtained the letter there, forged Cook's name on the cheque and appropriated the money. This bill would have been presented for payment the day that Cook died. On November 13th, 1855, Palmer owed Pratt, a money-lender, £11,500. That same day a horse of Cook's won at Shrewsbury races, and the owner received over £700 in bets on the race course, and was entitled to receive over £1,000 within a week. On that same 13th of November Pratt wrote to Palmer demanding immediate payment. Before the week was out Cook, who was staying at the "Talbot Arms," at Rugeley, just opposite Palmer's house, was dead, the money had gone from his pocket book, and his betting book had vanished. An illness of two days, marked by severe convulsions, had carried him off, and Palmer was accused of poisoning him by means of pills he administered and broth, made in his house, which he sent over to him at the "Talbot Arms." The poison supposed to be contained in these was strychnia, which it was proved Palmer had bought after Cook came to Rugeley, and the interest of the case turned on the likeness of the symptoms in strychnia poisoning to those of epilepsy and tetanus. On this likeness, joined to the fact that no strychnia was found in Cook's body when subjected to post-mortem analysis, though antimony was, the strength of the defence lay, and it is impossible to say how much it would have affected the verdict if Palmer's embarrassed circumstances and dishonest attempts to free himself at Cook's expense, had not shown that he had a powerful motive to influence him to crime. But as in his wife's body, which was exhumed and examined between his accusation and condemnation, traces of antimony were found, it is likely that, even if he had been acquitted of murdering Cook, he would have been condemned on another charge.

The cause of Cook's death was variously stated as apoplexy (by an aged and unsuspecting local practitioner who was called in, and who sent a box of pills to his patient by Palmer), epilepsy and tetanus. The suggestion of apoplexy was disposed of by the absence of any lesion of the brain that would have caused it; and epilepsy, by the fact that epileptic patients invariably lose consciousness during the fits, while Cook was conscious to the end. The suggestion of tetanus remained; the most difficult to deal with, from the difficulty of explaining exactly the difference between tetanic spasms and any other. Tetanus is familiarly called lockjaw, because one of the earliest symptoms is the rigidity of the muscles of the jaws and neck, producing difficulty in swallowing. Now, Cook had for some time suffered from sore throat, one of the results of an irregular life, and it was argued that this might produce tetanus. This, however, was positively denied by the medical experts. Moreover, tetanus does not begin as Cook's fatal illness did, with sudden and violent spasms. The early symptoms are milder, gradually developing into paroxysms of increasing frequency and severity; but there is no complete respite. In this, too, Cook's case did not run its course according to the habit of the disease. He suffered from violent sickness after taking some broth sent him by Palmer, and had a violent spasmodic attack during the night, when he had taken some pills supposed to come from Dr. Bamford, but brought by Palmer; and on recovering from this, was free from any similar attack for twenty-four hours, when a second and fatal one came on. Dr. Todd, physician to King's College Hospital, and a specialist in tetanus, contrasted the symptoms of strychnia poisoning with those of ordinary tetanus, very clearly. "The difference," he said, "between tetanus produced by strychnia and other tetanus is very marked. In the former case the duration of the symptoms is very short, and instead of being continuous in their development, they will subside if the dose has not been strong enough to produce death, and will be renewed in fresh paroxysms; whereas in other descriptions of tetanus the symptoms commence in a mild form, and become stronger and more violent as the disease progresses."

This evidence might not have sufficed to condemn Palmer in face of the fact that the analysts could find no strychnia in Cook's body, if it had not been that the accused's conduct at the post-mortem, at which he was present, had given the impression that he wished to prevent any minute examination of the contents of the body. He pushed Mr. Stevens, Cook's step-father, at the moment when a jar containing the viscera was being handed to him. When the jar had been covered with a double bladder and was standing ready to be taken away, Palmer removed it unnoticed from the table on which it stood to a place near the door, and when Mr. Stevens found it there, there was a slit in the covering. He also offered Myatt, a postboy, who was to drive Mr. Stevens and the jar to the station, £10 to upset the old man. This was all done before suspicion fell upon Palmer. Before the account of the analysis came from London an inquest had begun, and Palmer knew that he was in danger. He therefore tried to bribe the postmaster to open and show him the letter containing the results of the chemist's enquiry. Thus on every point the moral evidence went against him: His known need of money and the use he had made of Cook's name, his theft of the money in Cook's possession at the date of his death, his evident dread of the results of the post-mortem examination. It was this which condemned him, for the distinction between the symptoms of tetanus and strychnia is a fine one, perhaps fully to be appreciated only by a jury of scientists; and, indeed, though subsequent cases of strychnia have helped to elucidate its action since Palmer's day, it still remains one of the subtlest of poisons, and one which, in the hands of a clever criminal, may be long before it betrays him. But your murderer is usually foolish and rash, and when he has killed one or two victims with impunity he is apt to go on to yet another and another till he meets his doom.

